# Predictors of social leisure activities in older Europeans with and without multimorbidity

**DOI:** 10.1007/s10433-016-0375-2

**Published:** 2016-05-04

**Authors:** Henrike Galenkamp, Cristina Gagliardi, Andrea Principi, Stanislawa Golinowska, Amilcar Moreira, Andrea E. Schmidt, Juliane Winkelmann, Agnieszka Sowa, Suzan van der Pas, Dorly J. H. Deeg

**Affiliations:** 1 Department of Epidemiology and Biostatistics and the EMGO Institute for Health and Care Research, VU University Medical Center, De Boelelaan 1089a, 1081 HV Amsterdam, The Netherlands; 2 National Institute of Health and Science on Ageing (INRCA), Ancona, Italy; 3 Collegium Medicum Jagiellonian University, Krakow, Poland; 4 Institute of Labour and Social Studies, Warsaw, Poland; 5 Center for Social and Economic Research (CASE), Warsaw, Poland; 6 Institute of Social Science, University of Lisbon, Lisbon, Portugal; 7 European Centre for Social Welfare Policy and Research, Vienna, Austria

**Keywords:** Leisure activities, Aged, Chronic disease, Social participation

## Abstract

Older people spend much time participating in leisure activities, such as taking part in organized activities and going out, but the extent of participation may differ according to both individual and environmental resources available. Chronic health problems become more prevalent at higher ages and likely necessitate tapping different resources to maintain social participation. This paper compares predictors of participation in social leisure activities between older people with and those without multimorbidity. The European Project on Osteoarthritis (EPOSA) was conducted in Germany, UK, Italy, The Netherlands, Spain and Sweden (*N* = 2942, mean age 74.2 (5.2)). Multivariate regression was used to predict social leisure participation and degree of participation in people with and without multimorbidity. Fewer older people with multimorbidity participated in social leisure activities (90.6 %), compared to those without multimorbidity (93.9 %). The frequency of participation was also lower compared to people without multimorbidity. Higher socioeconomic status, widowhood, a larger network of friends, volunteering, transportation possibilities and having fewer depressive symptoms were important for (the degree of) social leisure participation. Statistically significant differences between the multimorbidity groups were observed for volunteering and driving a car, which were more important predictors of participation in those with multimorbidity. In contrast, self-reported income appeared more important for those without multimorbidity, compared to those who had multimorbidity. Policies focusing on social (network of friends), physical (physical performance) and psychological factors (depressive symptoms) and on transportation possibilities are recommended to enable all older people to participate in social leisure activities.

## Background

Increasing the level of participation of older people and promoting active ageing is an important goal of European policy in the context of current demographical ageing. Participation is a broad concept, but is often only viewed as engaging in activities that have economic value, such as labour market participation, volunteering or caregiving. However, many older people engage much more in other, more consumptive activities, such as going to a cultural event or on a day trip (Agahi et al. 2006; Klumb and Maier 2007; McKenna et al. 2007; Verbrugge et al. 1996). As these activities may increase their wellbeing and quality of life (Menec and Chipperfield 1997; Silverstein and Parker 2002), they constitute an important aspect of active ageing, according to the WHO’s definition (WHO 2002).

At the same time, many older people suffer from chronic health problems which increase the chance that participation is decreased or ceased altogether (Strain et al. 2002). Although a large body of research focuses on predictors of all kinds of social participation, little evidence exists on its predictors specifically in people with chronic health problems. People with health problems report that their health problems negatively affect their ability to engage in activities (Bowling 1995), and a substantial number report that they would like to do more activities in leisure time (Meulenkamp et al. 2013). Multimorbidity (the occurrence of two or more chronic diseases) is a rough and generic measure of health status, but with serious consequences for functioning and wellbeing (Marengoni et al. 2011). Given the increasing prevalence of chronic diseases among successive cohorts of older people (Crimmins and Beltrán-Sánchez 2011), and of multimorbidity, policies should focus on facilitating leisure participation particularly in this group.

The current paper focuses on social leisure activities, which can be defined as activities that are social in nature (i.e. it does not include individual activities such as reading or listening to music) and that are performed during free time and that are done by choice (Klumb and Maier 2007).

### Factors associated with participation in social leisure activities

Based on previous studies on predictors of leisure activity (Gagliardi et al. 2007; Strain et al. 2002), we expect a variety of factors, both individual and environmental, to be associated with older people’s participation in social leisure activities. While older people enjoy participating in social leisure activities as they age (Chen and Fu 2008), most studies revealed an age-associated decline in the number of activities people are engaged in, both in the general older population and in people with chronic disease (Janke et al. 2006; Lefrancois et al. 1997; van der Meer 2008; Zimmer et al. 1997).

An important explanation for decreasing participation with age can be found in the decline in health (Lefrancois et al. 1997; Strain et al. 2002). Older people in better physical and mental health show higher participation rates than those in poorer health. In particular, chronic diseases, functional limitations, depressive symptoms, poor perceived health and cognitive impairment are associated with lower participation in social leisure activities (Gagliardi et al. 2007; Griffin and McKenna 1999; Janke et al. 2006; Menec 2003; Nummela et al. 2008; Strain et al. 2002). In addition, in people who suffer from chronic diseases the severity of their condition, as indicated by ADL limitations or level of pain, might negatively influence leisure participation (Zimmer et al. 1997).

Other individual-level factors have been shown to be associated with a higher level of participation in social leisure activities as well. Among these were female gender (Gagliardi et al. 2007; Janke et al. 2006; Minhat and Mohd Amin 2012; van der Meer 2008), living with a partner (van der Meer 2008; van der Pas and Koopman-Boyden 2009), having a higher number of social contacts (Chen and Fu 2008; Menec 2003; Satariano et al. 2002), higher socioeconomic status (Gagliardi et al. 2007; Strain et al. 2002; van der Meer 2008) and psychological factors such as higher levels of self-efficacy (Perkins et al. 2008). One study that was performed specifically among older people with arthritis showed that those with a larger and more intimate network more often continued their participation in leisure activities, or replaced some activities with other activities, compared with those who had a smaller social network of relatives and friends (Zimmer et al. 1997).

Relatively little attention has been given to the environment of older people, which can be facilitating or restricting when it comes to participating in leisure activities. Thus, living in more prosperous neighbourhoods and having more transportation possibilities was shown to be associated with a higher level of participation in leisure activities (Dahan-Oliel et al. 2010; Gagliardi et al. 2007; Griffin and McKenna 1999; van der Meer 2008). In turn, the availability of public transportation and leisure opportunities may also depend on the level of urbanization. For example, in the Netherlands, living in a city is positively associated with participation in cultural recreation (van der Meer 2008).

Participating in other types of activities may pose restrictions to the time people may have at their disposal for social leisure activities. Previous studies showed that not providing informal care, being retired or working part-time were associated with higher participation in leisure activities (Janke et al. 2006). On the other hand, active older adults may engage in multiple activities such as leisure, caregiving and volunteering (Schmidt et al. 2015).

### Leisure participation in people with and without multimorbidity

According to Atchley’s *continuity theory* (Atchley 1989), older people attempt to preserve and maintain patterns of thoughts, activities and habits; and they prefer to accomplish this objective by using strategies tied to their past experiences of themselves and their social world. Atchley distinguishes internal and external continuity, where internal refers to the ‘self’ or ‘identity’. External continuity, in contrast, refers to the practice of interaction with familiar people and engagement in familiar activities. Indeed, it was found that previous participation in activities was an important predictor of current participation in leisure activities (Agahi et al. 2006; Strain et al. 2002; Verbrugge et al. 1996).

Our hypothesis following continuity theory is that, in the face of multimorbidity, people strive to continue their participation in social leisure activities. Despite this striving for continuity, however, many older people are not able to continue their participation in social leisure activities. The extent of participation is likely to differ according to both individual and environmental resources that are available. Continuity theory suggests that in order to maintain external continuity in the face of adversity, older people use familiar skills to do familiar things in familiar places in the company of familiar people. In addition, Atchley notes that an organizational and physical infrastructure is important for continuity in activities. Thus, we expect that a decrease in functional capacity in those with multimorbidity may increase the importance of other—e.g. socioeconomic, psychological or environmental—resources, even though these resources have similar characteristics for people with and without multimorbidity. Thus, the availability of public transport in one’s neighbourhood may become more important for people who are not able to walk long distances, and thus may be a more important factor for social leisure participation in those with multimorbidity (Martin et al. 2012). Likewise, people with multimorbidity have higher health care expenses and thus a low income level may be more detrimental for social leisure participation than for people without multimorbidity. Thus, we expect that the availability, or the lack, of individual and environmental resources become more important when health declines, and as such our hypothesis is that they are stronger predictors of social leisure participation in people with multimorbidity, compared with people without multimorbidity.

Most of the studies cited above were conducted in one specific city or country. The current study was performed in a multi-country study, and as such might be more representative for different regions in Europe. This is the first study that focuses on a wide range of possible predictors of participation in social leisure activities, and comparing these between people in good and poor health. Given the evidence for a broad range of predictors, our study includes as potential predictors socioeconomic status, social, environmental, psychological and factors of physical functioning. Country, age and gender will be taken into account as controls.

## Methods

The European Project on Osteoarthritis (EPOSA) involves six studies each performed in a different country with the same measurement instruments: Germany, UK, Italy, The Netherlands, Spain and Sweden. Random samples from these population-based samples are included, except for Italy, where a new sample was drawn. In each sample, 750 potential participants were contacted with the aim of recruiting 500 participants. Further details are available from the EPOSA design paper (van der Pas et al. 2013).

The overall age range was 65–85 years (with oversampling of the oldest respondents 80–85 years) in all countries except for the UK, which has an age range of 71–79 years. Data collection started between November 2010 and March 2011 in all countries, and ended between September and November 2011. Participants were visited in their homes by trained interviewers, except for Germany, Italy and Spain, where participants were examined by a trained interviewer in a health care centre and only disabled persons were visited in their home. The design and procedures of all six studies were approved by the Ethical Review Boards of the respective institutions.

The number of participants in EPOSA was 2942. A number of 125 respondents with missing data on participation in social leisure activities and 25 respondents without information on multimorbidity status were excluded from the analyses, resulting in 2792 participants. Respondents with missing data (*N* = 150) were more often women (*P* < .05) and had worse physical performance scores (*P* < .01). Other characteristics were not significantly different for these 150 respondents. The highest percentage of missing values on leisure activities (13.5 %) was observed for the UK and the lowest in Italy (1.9 %).

### Participation in social leisure activities

We used questions from the Maastricht Social Participation Profile (MSPP), which measures frequency and diversity of social participation both in a formal (organizational) and informal context, based on definitions of social participation by older people with a chronic disease (Mars et al. 2009). The following seven items comprised social leisure activities (out of nine that were included in EPOSA): ‘How often in the past four weeks have you (taken part in/been to) (1) a club, interest group or activity group, church or other similar activity? (2) a cultural or educational event such as the cinema, theatre, museum, talk or course (3) eaten out? (4) out to a pub, café or tearoom? (5) a public event? (6) an organised games afternoon or evening? For instance, bingo, quiz or card games. (7) a day trip organised by a club or society?’ Response categories ranged from 0 ‘not at all’ to 3 ‘more than twice a week’. An indicator of participation in social leisure (at least one of the items with a score ≥1) and a total social leisure activities score (range: 0–21) to measure the degree of involvement were used. Cronbach’s Alpha of these seven items of the MSPP was 0.59. The remaining two items of the MSPP represented volunteering and were included as predictors.

### Classification variable: Multimorbidity

Multimorbidity is the occurrence of two or more coexisting chronic conditions (van den Akker et al. 1996). The selection of chronic diseases that was explicitly investigated was based on their prevalence (>5 %). Participants were asked if they suffered from chronic diseases or symptoms that lasted for at least three months or diseases for which they had been treated or were followed by a physician. Included were chronic non-specific lung disease, cardiovascular diseases, peripheral arterial disease, stroke, diabetes, cancer, osteoarthritis and osteoporosis. Respondents with multimorbidity were compared with people reporting no or only one disease.

### Predictors of social and physical leisure participation

The selection of a broad range of predictors of participation was based on previous research among the general older population, *e.g.* (Gagliardi et al. 2007; Janke et al. 2006; van der Meer 2008).

Age and gender were included as *demographical variables*. As indicators of *socioeconomic status* were included self-reported income and educational level. Income was assessed by asking whether the respondent thinks the household is able to make ends meet with the total monthly income. This variable was dichotomized: with some or great difficulty versus easily or fairly easily. The highest level of education completed was coded as primary (elementary completed or not completed), secondary (vocational education or general secondary education) or tertiary (college or university education).

*Social* factors included marital status, categorized into single, widowed, divorced or having a partner (either married, registered partnership, cohabiting or living apart). Characteristics of the social network were assessed with the Lubben Social Network Scale (Lubben et al. 2006), assessing the number of family members and friends which respondents (1) see or hear from at least once a month, or (2) feel at ease with to talk about private matters, or (3) feel close to such that they could call on them for help. Responses ranged from 0 ‘none’ to 5 ‘9 or more’. Two subscale scores (separately for family and friends) were calculated (range 0–15). Cronbach’s alpha of the family and friends subscales was 0.81 and 0.80, respectively. Volunteer status was assessed with two questions from the MSPP, and was coded 1 if respondents carried out committee work for a club, society or other group or did any organised voluntary work.

It was asked whether respondents drive a car (‘yes’ vs. ‘no’). Environmental characteristics were measured using the Home and Community Environment instrument (White et al. 2010). The presence of the following features of the neighbourhood were assessed: parks and walking areas that are easy to use; places to sit and rest at bus stops, in parks, or other places where people walk; public facilities such as daily supermarket, bus stop, post office, bank or community centre; and public transportation close to home. For the first three features, response categories were dichotomized: a lot or some vs. not at all. For public transportation, the follow-up question to assess if respondents made use of these facilities was used, to make this question comparable to the question if participants drive a car. Urbanization grade was categorized as rural (<300 p/squared kilometre, <5000 inhabitants) or urban (>300 p/squared kilometre, >5000 inhabitants).

The following *psychological* variables were included. Anxiety and depressive symptoms were evaluated by the Hospital Anxiety Depression Scale (Zigmond and Snaith 1983), measuring levels of symptoms in the last week, comprising seven items for anxiety (Cronbach’s alpha 0.80, range 0–21) and 7 for depression (Cronbach’s alpha 0.71, range 0–21). Based on the finding that self-efficacy—the belief in one’s ability to execute certain behaviours—was related to participation in social leisure activities (Perkins et al. 2008), we expected that perceived control or mastery might also affect social leisure participation. In EPOSA, a measure of sense of mastery was included. The seven-item Pearlin Mastery Scale (Pearlin and Schooler 1978). consists of seven statements such as ‘I have little control over the things that happen to me’. The five response categories range from’strongly disagree to ‘strongly agree’, and higher sum scores indicate a higher sense of mastery (Cronbach’s alpha 0.78, range 0–28).

Indicators of *physical functioning* were self-reported disability and measured physical performance. Mobility and self-care disability problems were measured using two questions from the euroqol EQ-5D (Brooks et al. 2003). Respondents were categorized as limited (‘some problems’ or ‘confined to bed’) or not limited (‘no problems’). In addition, three performance tests were assessed (Guralnik et al. 1989): *walking speed* was measured by time taken to walk three metres as fast as possible, but not running; *repeated chair stands* by time taken to rise five times from a chair in normal tempo, without using the hands; and *standing balance* by the ability to perform the tandem stand for 10 s (with one foot behind the other and the heel of the first foot directly touching the toes of the other foot). The participants’ times for walking speed and repeated chair stands were divided into country-specific quartiles (scores 1–4; participants who were unable to perform these two tests were scored 0). The tandem stand is categorized into three groups. For comparability with the other performance tests, these three groups received the following scores: unable (<3 s  =  0), able to hold position for 3 to <10 s (2), and able to hold position for 10 s (4). Each of the three tests was scored from 0 (inability to carry out the test) to 4 (best performance), resulting in an overall performance score (range 0–12). For descriptive purposes, the physical performance score was dichotomized with scores higher than nine representing good performance, and scores lower or equal than nine representing poor performance.

### Statistical analysis

Age and sex distributions varied between the countries; therefore, a weighting variable was created for each individual within each country. The variables were derived from the European standard population in 2010 and calculated per sex and per 5-year age category, using the formula: *W* = *N*_exp_/*N*_obs_ (*N*_obs_ is the number of persons in a specific age/sex category in the country, and *N*_exp_ is the number of persons in a specific age/sex category in the European standard population in 2010). These were applied to all descriptive data with the exception of age and sex, allowing direct comparisons of the levels of participation across countries.

Bivariate associations were tested using T-tests or ANOVAs for continuous variables and Chi square tests for categorical variables. For descriptive purposes, network scores and mastery scores were dichotomized at the median value. For depressive and anxiety symptoms, existing cut-offs were used (Snaith 2003). Multivariate two-part regression models were applied: first, participation vs. non-participation was modelled, using logistic regression models. For the degree of participation in social leisure activities, linear regression models were applied. Those who did not participate were excluded from the analyses on participation frequency. All continuous variables were included as such and were not dichotomized. Average marginal effects (AMEs) are shown for the logistic models and standardized regression coefficients for the linear regression models. Significant differences were calculated through checking the AMEs and their standard errors, i.e. if AME_multimorbidity_ is larger/smaller than (AME_no multimorbidity_ ± 1.96*SE), the difference is significant at a p < .10 level.

All multivariate models to predict social leisure activities are stratified by multimorbidity status. In addition, models were run first without the indicators of physical functioning, disability and physical performance. These are considered to be in the pathway between multimorbidity and leisure participation (Verbrugge and Jette 1994) and, therefore, may mask any differences that may exist between the two groups. In a second model, the measures of physical functioning were included, in order to evaluate their contribution to the prediction of social leisure activities.

Analyses were conducted using IBM SPSS Statistics for Windows (Armonk, NY: IBM Corp) and STATA (College Station, TX: StataCorp LP). The level of statistical significance was set at *P* < .05.

## Results

Table 1 shows characteristics of the sample stratified by multimorbidity status. Almost fifty per cent of the sample had multimorbidity (48.7 %). Respondents with multimorbidity (N = 1358) were older, more often women, had lower socioeconomic status, and were more often widowed than people without multimorbidity (N = 1491). Fewer of them volunteered, and they had a smaller network of friends. More people with multimorbidity lived in urban areas and fewer of them drove a car, compared to people without multimorbidity. They also reported fewer parks and places to stop and rest in the neighbourhood. Finally, compared to people without multimorbidity, they were generally in poorer physical and psychological health.

**Table 1:** Sample characteristics: By multimorbidity status

	No multimorbidity *N* = 1434	Multimorbidity *N* = 1358	*P* ^1^	Unweighted *N*
	%/mean (sd)	%/mean (sd)		
Country				
Germany	14.7	11.8	<.001	2792
Italy	14.1	19.8		
Netherlands	20.0	21.5		
Spain	16.2	22.3		
Sweden	20.2	17.0		
UK	14.8	7.6		
Demographics				
Age (65-85)	73.4 (5.0)	75.0 (5.2)	<.001	2792
Female gender	45.7	57.4	<.001	2792
Socioeconomic status				
Primary education	39.3	51.5	<.001	2789
Secondary education	37.4	31.4		
Tertiary education	23.3	17.1		
Self-reported income: can easily make ends meet (vs. can not)	84.8	76.6	<.001	2755
Social				
With partner	69.4	63.1	<.001	2792
Divorced	6.0	5.0		
Widowed	18.5	26.5		
Single	6.1	5.4		
Network of family (0-15)	9.3 (3.2)	9.2 (3.3)	.211	2776
Network of friends (0-15)	7.9 (3.5)	7.3 (3.9)	<.001	2743
Volunteering	33.0	26.0	<.001	2790
Environmental				
Urban (vs. Rural)	60.5	64.6	.031	2789
Drives a car (yes vs. no)	69.3	56.0	<.001	2769
Uses public transport (yes vs. no)	56.4	53.1	.094	2734
Parks in neighbourhood (some/a lot vs. No)	88.9	85.1	.003	2777
Public facilities in neighbourhood (some/a lot vs. No)	88.8	88.6	.869	2772
Places to stop and rest (some/a lot vs. No)	85.5	82.5	.032	2757
Psychological health				
Depressive symptoms (0–20)	3.1 (2.8)	4.4 (3.4)	<.001	2728
Anxiety symptoms (0–20)	4.2 (3.4)	5.3 (3.9)	<.001	2729
Mastery (5–25)	19.8 (3.9)	18.5 (4.1)	<.001	2692
Physical health				
Mobility limitations (yes vs. no)	16.2	37.2	<.001	2771
Self-care limitations (yes vs. no)	5.6	13.9	<.001	2771
Physical performance (0–12)	8.8 (2.5)	7.5 (3.1)	<.001	2746
Chronic non-specific lung disease	3.3	25.1	<.001	2789
Cardiovascular diseases	8.9	43.6	<.001	2783
Peripheral arterial disease	1.9	22.5	<.001	2781
Diabetes mellitus	5.2	21.5	<.001	2786
Stroke	1.2	10.2	<.001	2777
Cancer	4.4	24.9	<.001	2790
Osteoarthritis	38.7	80.4	<.001	2786
Osteoporosis	3.7	30.7	<.001	2774
Social leisure activity				
Participation in social leisure activities (yes/no)	93.8	90.4	.001	2792
Degree of participation in social leisure activities (0-18)	5.0 (3.1)	4.4 (3.2)	<.001	2792

Weighted to the European standard population in 2010, except for age and gender

^1^Chi square test for categorical variables; *T* test for normally distributed variables

The share of people who reported their involvement in social leisure activities was quite high (Table 1, lower part): In those without multimorbidity, participation in social leisure activities was 93.8 %. Participation in those with multimorbidity was 90.4 %. Average scores on social leisure participation were 5.0 for people without multimorbidity, and 4.4 for those with multimorbidity. Table 2 shows the percentage who participated in each type of activity, by multimorbidity status. Largest differences in favour of the group without multimorbidity were observed in taking part in organised games and in day trips. Remarkably, the chance that people with multimorbidity had attended a public event was higher. Most predictor variables were associated with participation or the amount of participation in social leisure activities (Table 3). Only female gender, living in an urban area and having mobility limitations were not associated with being involved in social leisure activities. Variables not associated (in neither of the health groups) with the degree of participation were partner status and living in an urban area.

**Table 2:** Social leisure participation by multimorbidity status

How often in the past four weeks have you…^a^	No multimorbidity (<2 disease) (%)	Multimorbidity (≥2 diseases) (%)	*P* for difference
Taken part in a club, interest group or activity group, church or other similar activity?	54.9	51.7	.055
Been to a cultural or educational event such as the cinema, theatre, museum, talk or course?	42.7	35.7	<.001
Eaten out?	77.0	69.8	<.001
Been out to a pub, café or tearoom?	65.5	54.6	<.001
Been to a public event?	17.2	20.1	.030
Taken part in an organised games afternoon or evening? For instance, bingo, quiz or card games.	31.1	21.9	<.001
Been on a day trip organised by a club or society?	18.5	14.7	.005

^a^The percentages represent those who reported ‘less than once a week’, ‘once or twice a week’, or ‘more than twice a week’ versus those that did not take part in the activity

**Table 3:** Leisure activity by multimorbidity status and predictors

	Participation in social leisure							
	Participation (yes vs. no)				Degree of participation			
	No multimorbidity		Multimorbidity		No multimorbidity		Multimorbidity	
	%	P	%	P	Mean	P	Mean	P
Country								
Germany	98.0	<.001	97.4	0.002	6.4	<.001	6.1	<.001
Italy	87.1		88.1		3.9		3.6	
Netherlands	92.4		89.0		4.3		3.7	
Spain	91.5		87.7		4.7		4.2	
Sweden	95.3		90.6		4.6		4.4	
UK	98.0		97.0		6.2		6.3	
Demographics								
Age 65–74	95.0	0.019	93.7	<.001	5.1	0.007	4.7	<.001
Age 75–85	91.8		87.1		4.6		4.0	
Female gender	93.4	0.501	89.7	0.258	4.9	0.193	4.1	<.001
Male gender	94.3		91.6		5.1		4.8	
Socioeconomic status								
Primary education	90.2	<.001	87.0	<.001	4.3	<.001	3.8	<.001
Secondary education	95.5		93.2		5.1		4.4	
Tertiary education	97.2		95.5		5.8		5.8	
Income: can easily make ends meet	95.5	<.001	91.8	0.001	5.2	<.001	4.5	0.001
Income: cannot easily make ends meet	85.5		85.5		3.9		3.9	
Social								
With partner	93.6	0.709	90.3	0.036	4.9	0.781	4.3	0.707
Divorced	95.1		95.5		4.9		4.8	
Widowed	93.4		91.4		5.0		4.4	
Single	96.4		81.7		5.3		4.3	
Network score family ≤9	93.0	0.165	87.9	0.001	4.8	0.033	5.1	<.001
Network score family >9	94.8		93.2		5.1		4.7	
Network score friends ≤8	91.1	<.001	86.5	<.001	4.3	<.001	5.7	<.001
Network score friends >8	96.9		95.8		3.5		5.6	
Volunteering	98.0	<.001	98.8	<.001	6.4	<.001	6.3	<.001
Not volunteering	91.8		87.4		4.3		3.7	
Environmental								
Urban	93.6	0.724	90.8	0.432	4.9	0.572	4.3	0.601
Rural	94.1		89.5		5.0		4.4	
Drives a car	94.9	0.019	94.1	<.001	5.2	<.001	5.0	<.001
Does not drive a car	91.7		85.7		4.4		3.6	
Uses public transport	96.5	<.001	93.0	0.001	5.3	<.001	4.9	<.001
Does not use public transport	91.0		87.5		4.5		3.8	
Parks in neighbourhood	94.5	0.032	91.4	0.011	5.1	0.011	4.6	<.001
No parks in neighbourhood	90.1		85.6		4.4		3.2	
Public facilities in neighbourhood	94.6	0.006	91.2	0.004	5.0	0.103	4.5	<.001
No public facilities in neighbourhood	89.0		83.9		4.6		3.2	
Places to stop and rest	95.2	<.001	92.0	<.001	5.1	<.001	4.7	<.001
No places to stop and rest	86.8		84.1		4.0		3.2	
Psychological health								
High depression score (>7)	83.2	<.001	82.8	<.001	3.7	<.001	3.1	<.001
Low depression score (≤7)	94.9		92.5		5.1		4.7	
High anxiety score (>7)	88.6	<.001	87.9	0.024	4.3	<.001	3.9	<.001
Low anxiety score (≤7)	94.9		92		5.1		4.6	
High mastery score (>19)	96.2	<.001	92.1	0.199	5.3	0.001	4.9	<.001
Low mastery score (≤19)	91.3		90		4.7		4.0	
Physical health								
Mobility limitations	94.1	0.941	89.4	0.27	4.5	0.019	3.9	<.001
No mobility limitations	94.0		91.3		5.1		4.7	
Self-care limitations	93.5	0.868	83.3	<.001	4.0	0.003	4.5	<.001
No self-care limitations	94.0		91.9		5.0		3.4	
Physical performance score ≤9	92.8	0.067	89.0	0.004	4.8	0.004	4.1	<.001
Physical performance score >9	95.2		94.1		5.3		5.1	

Weighted to the European standard population in 2010

Table 4 shows the multivariate results for being involved in social leisure participation. Statistically significant predictors of participation in social leisure activities in those without multimorbidity were: self-reported income sufficiency, a large network of friends, volunteering, and the presence of places to stop and rest in the neighbourhood. In those with multimorbidity, significant predictors of participation in social leisure activities were secondary or tertiary versus primary education, being widowed, having a large network of friends, being a volunteer, driving a car, using public transport and having fewer depressive symptoms. Remarkably, having mobility limitations was associated with higher participation in social leisure activities. Compared with the dichotomous indicator of participation in social leisure activities, more similarities between the groups with and without multimorbidity were found in the degree of social leisure participation. The predictors higher education, being widowed and having fewer depressive symptoms were now also significant in the group without multimorbidity. Furthermore, anxiety symptoms significantly predicted a higher social leisure activities score in those without multimorbidity. Across multimorbidity groups, the strongest and the most consistent predictor appears to be whether someone is involved in volunteer work.

**Table 4:** Multivariate regression models, stratified by multimorbidity status: participation in social leisure activities and degree of participation in social leisure activities

	Participation in social leisure activities						Difference at *P* < .10	Degree of participation in social leisure activities						Difference at *P* < .10
	No multimorbidity AME (95 % CI)			Multimorbidity AME (95 % CI)				No multimorbidity B (95 % CI)			Multimorbidity B (95 % CI)			
Demographics														
Age (65–85)	−0.001	−0.003	0.002	−0.002	−0.005	0.002		−0.017	−0.052	0.018	0.014	−0.022	0.050	
Female gender	0.006	−0.023	0.035	0.002	−0.034	0.039		−0.090	−0.442	0.263	−0.099	−0.464	0.265	
Socioeconomic status														
Secondary vs. Primary education	0.017	−0.015	0.049	**0.044**	**0.004**	**0.084**		0.273	−0.116	0.663	0.027	−0.363	0.418	
Tertiary vs. Primary education	0.034	−0.013	0.081	**0.067**	**0.005**	**0.130**		**0.824**	**0.380**	**1.268**	**0.630**	**0.141**	**1.120**	
Self-reported income: can easily make ends meet (vs. cannot)	**0.039**	**0.010**	**0.068**	0.014	−0.021	0.049		**0.571**	**0.112**	**1.029**	−0.358	−0.769	0.053	*
Social														
Divorced vs. Partner	0.007	−0.054	0.067	0.023	−0.055	0.101		0.072	−0.597	0.740	0.524	−0.198	1.246	
Widowed vs. Partner	0.017	−0.018	0.052	**0.046**	**0.005**	**0.087**		**0.561**	**0.127**	**0.996**	**0.433**	**0.030**	**0.836**	
Single vs. Partner	0.005	−0.055	0.064	−0.044	−0.102	0.013		0.495	−0.198	1.189	0.695	−0.054	1.444	
Network of family (0–15)	0.000	−0.004	0.005	0.002	−0.003	0.007		0.006	−0.047	0.060	−0.003	−0.057	0.051	
Network of friends (0–15)	**0.006**	**0.002**	**0.010**	**0.006**	**0.002**	**0.011**		**0.165**	**0.116**	**0.213**	**0.179**	**0.132**	**0.226**	
Volunteering (yes vs. no)	**0.045**	**0.005**	**0.084**	**0.143**	**0.060**	**0.225**	*	**1.623**	**1.283**	**1.962**	**1.557**	**1.193**	**1.922**	
Environmental														
Urban (vs. Rural)	0.000	−0.030	0.030	0.000	−0.039	0.039		0.099	−0.308	0.505	−0.055	−0.468	0.359	
Drives a car (yes vs. no)	0.010	−0.022	0.041	**0.042**	**0.003**	**0.081**	*	0.166	−0.234	0.567	**0.513**	**0.117**	**0.909**	
Uses public transport (yes vs. no)	0.032	−0.002	0.067	**0.060**	**0.021**	**0.099**		**0.557**	**0.177**	**0.937**	0.354	−0.037	0.745	
Parks in neighbourhood (a lot or some vs. Not at all)	−0.006	−0.045	0.033	−0.011	−0.058	0.037		0.050	−0.538	0.638	0.304	−0.239	0.847	
Public facilities in neighbourhood (a lot or some vs. Not at all)	0.007	−0.030	0.043	0.009	−0.038	0.056		−0.152	−0.686	0.381	0.388	−0.178	0.954	
Places to stop and rest (a lot or some vs. Not at all)	**0.038**	**0.000**	**0.076**	0.037	−0.011	0.084		0.476	−0.098	1.051	0.226	−0.320	0.772	
Psychological health														
Depressive symptoms (0–20)	−0.003	−0.008	0.002	**−0.006**	**−0.011**	**−0.001**		**−0.092**	**−0.166**	**−0.018**	**−0.114**	**−0.178**	**−0.051**	
Anxiety symptoms (0–20)	0.002	−0.002	0.006	−0.001	−0.006	0.004		**0.066**	**0.007**	**0.125**	0.031	−0.025	0.087	
Mastery (5−25)	0.002	−0.002	0.006	−0.004	−0.009	0.001		−0.002	−0.056	0.052	0.006	−0.046	0.058	
Physical health														
Mobility limitations (yes vs. no)	0.026	−0.012	0.064	**0.047**	**0.006**	**0.089**		0.230	−0.249	0.709	−0.043	−0.444	0.358	
Self-care limitations (yes vs. no)	0.016	−0.037	0.069	−0.037	−0.084	0.011		−0.606	−1.341	0.129	0.254	−0.302	0.811	
Physical performance (0–12)	0.002	−0.003	0.007	0.004	−0.002	0.010		0.035	−0.039	0.108	**0.097**	**0.031**	**0.163**	

* Bold coefficients indicate a significance level of * P* < 0.05

*AME* average marginal effect; b, regression coefficient

Adjusted for country

**Table 5:** Multivariate regression models of social leisure activities, stratified by multimorbidity status, without measures of physical functioning

	Participation in social leisure activities						Difference at *P* < .10	Degree of participation in social leisure activities						Difference at *P* < .10
	No multimorbidity AME (95 % CI)			Multimorbidity AME (95 % CI)				No multimorbidity B (95 % CI)			Multimorbidity B (95 % CI)			
Demographics														
Age (65–85)	−0.001	−0.003	0.002	−0.002	−0.006	0.001		−0.020	−0.054	0.013	−0.001	−0.035	0.033	
Female gender	0.005	−0.024	0.033	0.002	−0.035	0.039		−0.113	−0.462	0.236	−0.113	−0.473	0.248	
Socioeconomic status														
Secondary vs. Primary education	0.022	−0.010	0.053	**0.045**	**0.005**	**0.084**		0.279	−0.106	0.664	0.048	−0.341	0.437	
Tertiary vs. Primary education	0.036	−0.011	0.083	**0.068**	**0.004**	**0.132**		**0.792**	**0.354**	**1.230**	**0.677**	**0.192**	**1.163**	
Self-reported income: can easily make ends meet (vs. cannot)	**0.039**	**0.010**	**0.067**	0.015	−0.020	0.051		**0.553**	**0.101**	**1.006**	−0.356	−0.763	0.051	*
Social														
Divorced vs. Partner	0.007	−0.054	0.068	0.027	−0.051	0.106		0.097	−0.565	0.759	0.457	−0.262	1.177	
Widowed vs. Partner	0.014	−0.021	0.048	**0.046**	**0.006**	**0.087**		**0.522**	**0.093**	**0.952**	0.386	−0.014	0.785	
Single vs. Partner	0.006	−0.054	0.065	−0.043	−0.101	0.014		0.379	−0.302	1.059	0.685	−0.057	1.426	
Network of family (0–15)	0.000	−0.004	0.004	0.003	−0.003	0.008		0.007	−0.045	0.060	−0.007	−0.060	0.047	
Network of friends (0–15)	**0.006**	**0.003**	**0.010**	**0.007**	**0.002**	**0.011**		**0.158**	**0.111**	**0.206**	**0.185**	**0.138**	**0.231**	
Volunteering (yes vs. no)	**0.047**	**0.007**	**0.087**	**0.148**	**0.065**	**0.231**	*	**1.626**	**1.290**	**1.963**	**1.600**	**1.239**	**1.962**	
Environmental														
Urban (vs. Rural)	0.004	−0.026	0.033	0.001	−0.038	0.040		0.125	−0.276	0.526	−0.073	−0.482	0.337	
Drives a car (yes vs. no)	0.010	−0.021	0.041	**0.046**	**0.008**	**0.085**	*	0.169	−0.226	0.564	**0.611**	**0.224**	**0.999**	*
Uses public transport (yes vs. no)	0.033	−0.001	0.066	**0.055**	**0.017**	**0.094**		**0.593**	**0.215**	**0.971**	**0.406**	**0.022**	**0.791**	
Parks in neighbourhood (a lot or some vs. Not at all)	−0.001	−0.039	0.036	−0.015	−0.062	0.032		0.009	−0.571	0.588	0.280	−0.255	0.814	
Public facilities in neighbourhood (a lot or some vs. Not at all)	0.008	−0.028	0.044	0.014	−0.032	0.061		−0.152	−0.683	0.379	0.388	−0.174	0.949	
Places to stop and rest (a lot or some vs. Not at all)	0.031	−0.006	0.068	0.038	−0.010	0.086		0.475	−0.090	1.040	0.217	−0.325	0.759	
Psychological health														
Depressive symptoms (0–20)	−0.003	−0.008	0.002	**−0.006**	**−0.011**	**−0.001**		**−0.098**	**−0.171**	**−0.025**	**−0.118**	**−0.180**	**−0.055**	
Anxiety symptoms (0–20)	0.002	−0.002	0.006	0.000	−0.005	0.005		**0.063**	**0.005**	**0.122**	0.037	−0.018	0.092	
Mastery (5–25)	0.002	−0.002	0.006	−0.003	−0.008	0.001		0.002	−0.052	0.055	0.022	−0.028	0.073	

* Bold coefficients indicate a significance level of * P* < 0.05

*AME* average marginal effect, *b* regression coefficient

Adjusted for country

Statistically significant differences between predictors in the groups with and without multimorbidity are shown with an asterisk, only if one of the two AMEs are statistically significant. Only three predictors differed statistically significant between multimorbidity groups. Volunteering and driving a car were more important for participating in the group with multimorbidity. Self-reported income was more important for the degree of participation in those without multimorbidity. Differences between AMEs were also observed for mastery, public facilities and for self-care limitations, but in neither of the health groups, these were statistically significant predictors of participation or the degree of participation.

Models that were not adjusted for indicators of physical functioning (mobility and self-care limitations and physical performance) revealed rather similar results, except for ‘places to stop and rest’ and being widowed, which were no longer statistically significant predictors of participation or the degree of participation. In addition, using public transport was now also a significant predictor of the degree of participation in those with multimorbidity. Driving a car was more important for those with multimorbidity, now not only for participation but also for the degree of participation (Table 5).

## Discussion

This study examined the predictors of leisure participation in older European people with and without multimorbidity. As expected, older people with multimorbidity participated less in social leisure activities. In addition, they participated at a lower frequency compared to those without multimorbidity. Participation in social leisure activities was determined by factors in all the domains studied, including socioeconomic, social, environmental, and psychological and physical functioning characteristics.

Following Atchley’s continuity theory (Atchley 1989), we hypothesized that people with multimorbidity strive to continue their participation in social leisure activities, but that the importance of other resources than health—for example, socioeconomic, psychological or environmental resources—becomes greater than that for people without multimorbidity. This hypothesis was partly confirmed: both driving a car and volunteering were more important predictors in older people with multimorbidity, compared with those with no multimorbidity. In general, those with multimorbidity were older, reported more disability, and had fewer psychosocial resources. This might explain the importance of driving a car for their participation. Contrary to our hypothesis, having sufficient income to make ends meet was a more important predictor for people without multimorbidity. It seems that for those who are not particularly restricted in social leisure participation for health reasons, other factors—such as insufficient income—are important barriers for social leisure participation.

The strongest and most consistent predictor of participation in social leisure activities was being active in volunteer work. Since this was a cross-sectional study, the direction and causality of reported associations remain unclear. Thus, a person’s participation in volunteering may increase their participation in social leisure activities, presumably by an increased level of social ties (Pilkington et al. 2012) and vice versa. In addition, there may be overlap between the two, since volunteering may occur within the measured domains of social leisure. Since almost all volunteers participated in social leisure activities, we also ran models excluding volunteering as a predictor, but these models yielded very similar results with respect to the other predictors. This implies that findings with regard to the other predictors were not influenced by the strong association between volunteering and social leisure participation.

Higher education, widowhood, a large network of friends, using public transport and fewer depressive symptoms were equally important predictors in both health groups, although some appeared significant in only one of the groups. These predictors were found for participation as well as for the degree of participation. The importance of higher education and perceived income confirms results by other studies (Gagliardi et al. 2007; Strain et al. 2002), and may be related to the costs of some social leisure activities, such as visiting restaurants and cultural events. Being widowed appeared predictive for social leisure participation in both health groups. Also, having mobility limitations was predictive for social leisure participation in the group with multimorbidity. In these cases, it might be that people engage in social leisure activities to avoid isolation caused by the loss of the partner and reduced mobility. Some activities (e.g. organised games afternoon or evening) could be performed at home, where mobility limitations are not necessarily a barrier. We observed that people with multimorbidity more often attended public events, which was a counterintuitive finding. Since many people were active in volunteer work, we examined if volunteering competed with attending public events in those without multimorbidity. But it appeared that among the volunteers, people with multimorbidity were even more likely to attend public events. Another tentative explanation might be that people with multimorbidity attend more disease or health-related events, but this hypothesis needs further studying.

Gagliardi et al. (2007) showed that being dependent on public transport was associated with engagement in more at-home activities, but their study did not focus specifically on people with poor health. Transportation possibilities may compensate for mobility limitations, and this might explain why public transportation was a significant predictor of (the degree of) participation in social leisure activities, both in people with and without multimorbidity.

With regard to depressive symptoms, the association found may also represent two directions: a lack of social interaction may increase depressive symptoms; and depressive symptoms may have a negative influence on social leisure participation. Interventions targeted at depressed individuals include the prescription of structured exercise (Bridle et al. 2012), which are also considered a type of leisure activity. Thus, increasing other types of leisure activities may have important mental health benefits for older people, and in turn, benefits for social functioning.

With respect to the network of friends, research on changes in older people’s networks showed that the network may still increase, more often in younger than in older olds (van Tilburg and Broese van Groenou 2002). However, in the face of physical decline, the proportion of friends in the network has been shown to decrease (Aartsen et al. 2004). Thus investing in a large network of friends seems important especially for younger olds with multimorbidity.

The results of this study partly correspond to those of several previous studies that have assessed predictors of social leisure activities in the general older population, but differences with regard to specific predictors were also observed (Chen and Fu 2008; Gagliardi et al. 2007; Janke et al. 2006; van der Meer 2008). Studies vary greatly in their conceptualization of leisure participation. Groups of activities were for example based on factor analysis (Gagliardi et al. 2007; Lennartsson and Silverstein 2001; Silverstein and Parker 2002), or defined by the extent to which activities are social (Menec 2003), formal or informal (Broese van Groenou and Deeg 2010; Janke et al. 2006). The inclusion of multiple countries and a broad set of social, health and psychosocial predictors might explain differences with these previous studies.

Some of our findings might inform policies that aim at improving older people’s participation in leisure activities. In both the multimorbidity and non-multimorbidity groups, only a few people in each country did not engage in social leisure activities at all. As the participation rates in both health groups were quite high, we included also the degree of participation. This measure revealed a larger difference in participation between groups with and without multimorbidity. This indicates that interventions are better aimed at increasing the amount or promoting the continuation of leisure participation, rather than the initial decision to participate. Interestingly, predictors of the degree of participation were more similar between the groups with and without multimorbidity. This suggests that policy measures to increase the degree of social leisure activity should not necessarily be different for people with and without multimorbidity. This study gives some indications as to which personal and environmental factors should be targeted at in order to increase participation in social leisure activities in older people, namely good physical and psychological health, a large network of friends and appropriate transportation possibilities.

This study included multiple indicators of older people’s physical environment that might influence their participation in leisure activities. Some associations emerged that need further investigation. For example, the availability of places to stop and rest in the neighbourhood was associated with social leisure participation only in those without multimorbidity, even though mobility barriers are reported often by people with chronic diseases (Martin et al. 2012; Theis and Furner 2011). Our data show that a characteristic of the environment that might increase the social leisure participation of older people is the availability and accessibility of public transport. The question used for assessing use of public transport was limited to those who reported the availability of public transportation in the neighbourhood. The twelve percent who did not have public transportation available, however, might have used it for example while being in a different neighbourhood. It should be addressed in future research whether improving the availability of transport increases the level of participation in leisure activities.

A limitation of this study is non-response to certain variables in the study, which was related to physical performance. This influences the representativeness of our findings, and should be kept in mind when applying our results to the general older European population. The influence of country differences in non-response on our findings remains uncertain. In this study, different types of social leisure activities were combined, but the effect of leisure participation on wellbeing likely depends on the degree to which activities match older people’s preferences (Herzog et al. 1991). Further research might address differences in leisure preferences between people with and without multimorbidity.

We acknowledge that other meaningful groups with different health statuses can be made. For example, having more than two diseases, or multimorbidity combined with disability, might negatively affect participation in social leisure activities. Since this is a first step in exploring whether determinants of social leisure activity differ by health status, we decided to use multimorbidity—with proven negative effects on wellbeing and functioning (Marengoni et al. 2011)—as a grouping factor. This study did not focus on the specific diseases people with multimorbidity have, and future studies may address diseases with various types of disability, to provide more specific recommendations for interventions that enable people with chronic diseases to stay socially active. Eighty per cent of those with multimorbidity in this study reported to have osteoarthritis, a leading cause of disability among older people, especially when multimorbid conditions are present (CDC 2013). The high prevalence of arthritis may explain why better physical performance predicted social leisure activities. This high prevalence, however, cannot explain why mobility limitations predicted participation in social leisure activities, a finding that is difficult to explain.

No information on personality characteristics was available for this study, while personality aspects may play an important role in social leisure participation, and the degree of participation. In addition, no information on previous participation was available in this study, while this has been shown to be strong predictor of current participation (Agahi et al. 2006). It remains to be seen in future research if this continuity holds equally for older people with and without good health. Finally, it was beyond the scope of this article to include in the analyses country-level information, such as the availability of organized social and physical activities and the differences in climate. We acknowledge that there were quite some differences in leisure participation between the six countries, and future studies may examine if the factors that have been identified in our study are important predictors in each of the countries.

To conclude, this study conducted in a sample of older Europeans showed that participation in leisure activities is rather high. This is good news with respect to the current focus of European policies on active ageing. However, (the amount of) participation in social leisure activities may be increased in particular in those with multimorbidity. They participated less, and less frequently in social leisure activities compared to older people without multimorbidity. To increase their wellbeing and quality of life, policies should focus on social (network of friends), physical (physical performance) and psychological factors (depressive symptoms), in order to increase social leisure participation. In addition, transportation possibilities may be improved for both groups of older people, as these were important predictors of participation in social leisure activities.
